# 29ièmes Journées Franco-Belges de Pharmacochimie: Meeting Report

**DOI:** 10.3390/ph8040758

**Published:** 2015-11-17

**Authors:** The Members of the Organizing Committee

**Affiliations:** 1 Raphaël Frédérick: Medicinal Chemistry Research Group (CMFA), Louvain Drug Research Institute (LDRI), Université Catholique de Louvain, Woluwé-Saint-Lambert 1200, Belgium; E-Mail: raphael.frederick@uclouvain.be; 2 Lionel Pochet: Department of Pharmacy, Namur Medicine & Drug Innovation Center (NAMEDIC), Namur Research Institute for Life Sciences (NARILIS), University of Namur, 61, Rue de Bruxelles, B-5000 Namur, Belgium; E-Mail: lionel.pochet@unamur.be; 3 Pascal De Tullio: Center for Interdisciplinary Research on Medicines (CIRM), University of Liege, Liège 4000, Belgium; E-Mail: P.DeTullio@ulg.ac.be; 4 François Dufrasne: Laboratoire de Chimie Pharmaceutique Organique, Faculté de Pharmacie, Université Libre de Bruxelles Campus Plaine CP 205/5, Brussels 1050, Belgium

**Keywords:** medicinal chemistry, pharmacochemistry, pharmacology, screening, target discovery, target validation

## Abstract

The “Journées Franco-Belges de Pharmacochimie” is a recognized two-day annual meeting on Medicinal Chemistry that is renowned for the advanced science presented, conviviality, and outstanding opportunities for senior and young scientists to exchange knowledge. Abstracts of plenary lectures, oral communications, and posters presented during the meeting are collected in this report.

## 1. Aim and Scope of the Meeting

The “Journées Franco-Belges de Pharmacochimie” (JFB) is a widely recognized annual meeting on Medicinal Chemistry. This two-day symposium aims to promote exchanges between medicinal chemists mainly from France and Belgium. It is renowned for the advanced science presented, conviviality, and outstanding opportunities for senior and young scientists to exchange knowledge.

The scientific program included one tutorial lecture and four plenary lectures by internationally recognized scientists. An important part of the program was devoted to open lectures (10 oral communications) giving the opportunity for young scientists to present their research. The themes discussed during the meeting were those generally encountered by medicinal chemists: organic synthesis, bioinformatics and computer-aided drug design, pharmacological tests and molecular biology. This year the official language for the lectures was English.

## 2. Conferences

### 2.1. Efficient Access to Novel Mono- and Disubstituted Pyrido[3,2-d]pyrimidines

Sylvain Routier

Institut de Chimie Organique et Analytique, Univ Orléans, CNRS, UMR 7311, B.P. 6759, 45067 Orléans Cedex 2, France; E-Mail: sylvain.routier@univ-orleans.fr

Polynitrogen heterocycles such as diazines are widely used in the design of biologically active structures. Recently, the quinoxaline heterocycle was included in anticancer selective kinase inhibitors acting at the ATP binding site. This strategy led to ZD 1839 (gefitinib, Iressa^®^) and PD 173074, which both exhibited strong antiangiogenic activities.

Pyridopyrimidines, nitrogen isosteres of the previously evocated heterocycles, were claimed to have medicinal applications too. In particular, the recent development of anticancer agents led to some pyrido[2,3-d]pyrimidines analogs like PD 166285 and VK-19911, which were described as very promising PDGF and MAP kinase inhibitors, respectively.

For the reason given above, the synthesis of pyrido[3,2-d]pyrimidine derivatives provides an interesting challenge. In this work, we described the novel method to obtain the di- and tri-chloropyrido[3,2-d]pyrimidines. Exploration of the reactivity was performed prior to the design of kinase inhibitors. Previously unknown methodologies were next used to design two series of bioactives molecules able to target kinases involved in SNC diseases (CDK5) or in oncology (dual PI3k/mTor).


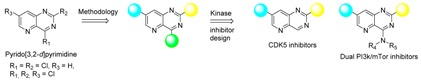


### 2.2. Design, Synthesis and Evaluation of Th1 Skewing a-GalCer Analogues

Serge Van Calenbergh

Laboratory for Medicinal Chemistry (FFW), Ghent University, Ottergemsesteenweg 460, Ghent B-9000, Belgium; E-Mail: Serge.VanCalenbergh@UGent.be

Invariant NKT (iNKT) cells represent a unique subset of T lymphocytes that have an important regulatory role in the protection against tumour cells, auto-immune diseases and certain infections. iNKT cells recognize the prototypical ligand α-galactosylceramide (α-GalCer), presented by CD1d.

This presentation will focus on our attempts to design α-GalCer analogues that polarize the cytokine response towards Th1. Towards this end we carefully investigated modifications at two distinct sites of α-GalCer.

First, the ability of analogues modified at the 6′′-position of the galactose ring to induce a polarized Th1 response will be demonstrated. Crystallographic studies indicated that two lead compounds, *i.e.*, NU-α-GalCer (**1**) and PyrC-α-GalCer (**2**), undergo additional interactions with either CD1d or the T-cell receptor (TCR) of iNKT cells. Both cases illustrate a unifying concept that judiciously chosen modifications of α-GalCer’s carbohydrate moiety may enhance the stability of the CD1d-glycolipid-TCR complex and lead to a more robust induction of Th1 cytokines in mice, while retaining activity in human iNKT cells.


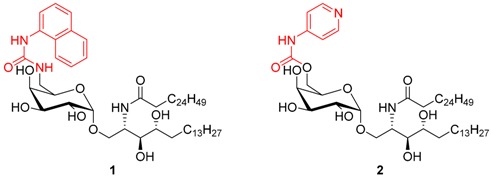


In a second part two synthetic approaches to explore if introduction of aromatic groups in the aliphatic part of the sphingosine moiety may be accommodated by the F’-pocket, enhance binding to CD1d and lead to an improved cytokine profile will be discussed. Preliminary biological results will be reported.

### 2.3. Tackling a New Paradigm in Drug Discovery: The Case of Poly-Pharmacology

Antonio Macchiarulo

Department of Pharmaceutical Sciences, University of Perugia, Via del Liceo 1, Perugia 06123, Italy; E-Mail: antonio.macchiarulo@unipg.it

The awareness that drugs often bind to more than one target has elbowed its way into the scientific community, ushering in the paradigm of polypharmacology in drug discovery (Hopkins A.L. *Nature* 2009, *462*, 167–168). Although this aspect has commonly been considered as undesirable promiscuity responsible for unwanted side effects, in many cases it is a key component to the therapeutic efficacy of drugs (Keiser M.J., *et al.* *Biochemistry* 2010, *49*, 10267–10276). Knowledge on polypharmacology can therefore help explain why some drugs work better than expected, or why other drugs have diverse side effects, albeit acting on the same target. Polypharmacology is the result of poor ligand specificity that combines with protein promiscuity (Macchiarulo, A., *et al.* *Nat. Biotechnol.* 2004, *22*, 1039–1045). In this framework, both protein-based and ligand-based computational techniques are being developed to infer about polypharmacology of ligands (Boran, A.D., *et al.* *Curr. Opin. Drug Discov. Devel.* 2010, *13*, 297–309).

In this communication, two case studies are presented. The first study shows a computational approach aimed at investigating the aspects of polypharmacology in the superfamily of human nuclear receptors (NRs). Specifically, a protein-based approach will be discussed as instrumental in charting key components and interactions of NR binding sites, with the aim of aiding the rationalization and optimization of selectivity and/or multi-target profile of selected NR ligands (Macchiarulo, A., *et al.* *Med. Chem. Commun.* 2013, *4*, 216–227). In the second case study, complementary ligand-based approaches are discussed as research tools to investigate the occurrence of ligand polypharmacology in the superfamily of poly(ADP-ribosyl)transferases (PARPs) (Wahlberg, E, *et al.* *Nat. Biotechnol.* 2012, *30*, 283–288, and Marchand, J.R., *et al.* *Biochim. Biophys. Acta* 2014, *1844*, 1765–1772).

### 2.4. Analgesic Drugs Targeting PDZ Proteins and TREK Channels

Sylvie Ducki

Université Clermont Auvergne, ENSCCF, Institut de Chimie de Clermont-Ferrand, BP 10187, Aubiere F-63174, France; E-Mail: sylvie.ducki@ensccf.fr

Pain affects nearly 70 million people on the seven principal world markets, causing a great degree of discomfort among patients and an enormous economic and social burden. Inadequate pain control and side-effects associated with current analgesics has encouraged the development of original analgesics with novel modes of action to address the unmet needs of patients.

PDZ domains are involved protein–protein interactions and are almost always associated with the cell membrane where they play an essential role in the clustering of proteins and signal transduction. Disrupting interaction between PDZ-containing protein PSD-95, and its natural ligand 5-HT2A receptor, was found to reduce hyperalgesia in a rodent model of neuropathic pain. Thus, inhibiting this interaction could lead to the development of a novel class of analgesic agents.

Amongst the mechanisms of action involved in pain transmission and perception, TWIK-Related K+ channel, TREK-1, has recently emerged as an attractive therapeutic target for the development of a novel class of analgesic drugs. It has been reported that TREK-1 −/− mice were more sensitive than wild-type mice to painful stimuli, suggesting that activation of TREK-1 could result in pain inhibition.

## 3. Short Oral Communications

### 3.1. Design, Synthesis and Activity Evaluation of New Irreversible Myeloperoxidase Inhibitors Derived from Benzodioxole

Jalal Soubhye ^1,^*, Iyas Aldib ^1^, Bénédicte Valet ^1^, Sarra Tadrent ^1^, Michel Gelbcke ^1^, Paul G. Furtmüller ^2^, Jean. Nève ^1^, Christian. Obinger ^2^, François. Dufrasne ^1^ and Pierre Van Antwerpen ^1^

^1^ Laboratoire de Chimie Pharmaceutique Organique, Faculté de Pharmacie, Faculty of Pharmacy, Université Libre de Bruxelles, Brussels 1050, Belgium

^2^ Department of Chemistry, Division of Biochemistry, Vienna Institute of BioTechnology, BOKU – University of Natural Resources and Life Sciences, Vienna 1000, Austria

* Author to whom correspondence should be addressed; E-Mail: jal.sub@hotmail.com.

The essential role of MPO in the immunity system is the oxidation of the pathogenic agents inside the neutrophils. In some cases, this enzyme causes oxidative damages for the host tissues contributing in the development of inflammatory syndromes. Thus, the inhibition of MPO in the circulation can be useful in the treatment of several inflammatory diseases. In the last decade, we described some potent reversible MPO inhibitors derived from fluorotryptamine (Soubhye, J., *et al.* *J. Med. Chem.* 2010, *53*, 8747–8759; Soubhye, J., *et al. J. Med. Chem.* 2013, *56*, 3943–3958). In addition we have reported that the SSRI agent (paroxetine) can irreversibly inhibit MPO at low nanomolar range (Soubhye, J. *et al. J. Pharm. Pharmacol.* 2014, *66*, 1122–1132). With the docking experiments, the important chemical groups in both paroxetine and fluorotryptamine derivatives were determined and general structure of the new series was designed.


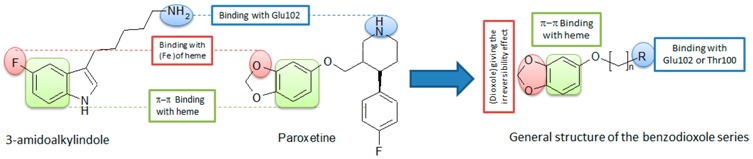


After determination of the general structure, several modifications, including the length of the side chain, the functional group, the aromatic ring and the dioxole group, were applied to study the SAR of this series. Docking experiments for these designed compounds indicated that the length of the side chain must be between three and six carbons, the functional groups must be amine or amide, the best aromatic group is benzene and two hydroxyl groups give the compound an interaction with active site higher than dioxole. These compounds were synthesized and tested *in vitro* by a taurine chloramine test in order to determine the IC_50_ values. The results confirmed the docking test for several compounds. It is found that the IC_50_ of the compounds with amine are the lowest values among all the functional groups (IC_50_ = 10–60 nM). It is shown also that five carbons on the side chain gives the best activity for the compounds with amine while those of amide the best activity was in the compound with three carbons. Unlike the docking, the *in vitro* test showed that the compound with two OH groups instead of a dioxole hasn’t any activity. Kinetic studies showed that all of the active compounds reduced the Compound I of MPO to the inactive form Compound II in very fast way. Then the inhibitor reacts irreversibly with the enzyme causing destruction of the heme. The compound with two OH moieties reacts with both Compounds I and II in very fast way to regenerate the native enzyme which in turn is oxidized to Compound I that produces HOCl (the active oxidative molecule of MPO).

### 3.2. Design of Novel α7 nAchR Tracers: from Ligand Synthesis to 18F PET Imaging Evaluation

Liliana Boiaryna ^1,^*, Nuno Rodrigues ^1^, Emilie Bertrand ^1^, Aziz Ouach ^1^, Frédéric Pin^1^, Johnny Vercouillie ^2^, Sylvie Mavel ^2^, Zuhal Gulhan^2^, Gabrielle Chicheri ^3^, Jean-Bernard Deloye ^3^, Denis Guilloteau ^3^, Franck Suzenet ^1^, Sylvie Chalon ^2^ and Sylvain Routier ^1^

^1^ Institut de Chimie Organique et Analytique, Université d’Orléans, UMR CNRS 7311, Orléans 45100, France

^2^ UMR Inserm U930, Université François Rabelais, Tours 37000, France

^3^ Laboratoires Cyclopharma, Tours 37000, France

* Author to whom correspondence should be addressed; E-Mail: liliana.boiaryna@univ-orleans.fr.

The neurotransmitter acetylcholine exerts its effects on the central nervous system through two distinct muscarinic mAChR and nicotinic nAChR receptor types. (a) Due to distribution and abundance of nAchR receptors in the hippocampus and cortex enable diagnosis and therapy of some brain disorders which affect these cerebral regions (Wu, J., *et al.* *Int. J. Alzheimers Dis.* 2010, *2010*, 548913 ; Dajas-Bailador, F., *et al.* *Trends Pharm. Sci.* 2004, *5*, 317–324; Gotti, C., *et al.* *Trends Pharm. Sci.* 2006, *27*, 482–491; Paterson, D. *et al.* *Prog. Neurobiol.* 2000, *61*, 75–111). Namely, α7 nAChR agonists were identified and allowed the design of novel therapeutic agents for Alzheimer Disease (AD). In other hand, a human compatible [^18^F]-labeled PET tracer for early diagnostic or validation of therapy efficiency of AD is indubitably crucial. In this aim, we envisioned to design novel α7 nAChR ligands as potential candidates for [^18^F] PET tracers design.

Based on our expertise in heterocyclic bio-mimetic development, (b) we obtained a wide library of novel α7 nAChR ligands, containing quinuclidine, tropane or 8H-quinolizine moiety (Neagoie, C., *et al.* *Eur. J. Med. Chem.* 2012, *49*, 379–296; Boulahjar, R., *et al.* *J. Med. Chem.* 2012, *55*, 9589–9606). SAR evaluation proved high range of affinity going to 0.12 nM. The most promising ligands were radio-labeled by incorporating of ^18^F by nucleophilic substitution of appropriate precursors. Further *in vivo* studies in rats showed structure-dependant results concerning the passage through blood-brain barrier and the accumulation ratio to cerebellum. We present herein chemistry, (c) SAR studies, molecular modeling docking studies which confirmed the binding mode of the developed ligands, *in vitro* efficiency (SAR), radio-labeling and *in vivo* results in rats (Chalon, S. WO 2012143526, 26 October 2012; Pin, F., *et al.* *Eur. J. Med. Chem.* 2014, *82*, 214–224). We gratefully acknowledge the financial support of this work provided by COSMI FEDER, Region Centre and Labex IRON.

### 3.3. Design of New Selective Small-Molecular-Weight Inhibitors of FXIIa

Charlotte Bouckaert ^1,^*, Silvia Serra ^1^, Jean-Michel Dogné ^1^, Raphaël Frédérick ^2^ and Lionel Pochet ^1^

^1^ Department of Pharmacy, Namur Medicine & Drug Innovation Center (NAMEDIC), Namur Research Institute for Life Sciences (NARILIS), University of Namur, 61, Rue de Bruxelles, B-5000 Namur, Belgium

^2^ Medicinal Chemistry, Research Group (CMFA - LDRI), Université Catholique de Louvain (UCL), Bruxelles 1200, Belgium

* Author to whom correspondence should be addressed; E-Mail: charlotte.bouckaert@unamur.be.

Background: Thrombotic diseases are a major cause of morbidity and mortality in the industrialized world. Anticoagulants have proven their efficacy to address these disorders but they are still associated with several drawbacks. In the quest of developing the ideal anticoagulant, the inhibition of activated coagulation factor XII (FXIIa) emerges as an attractive strategy for the development of safe antithrombotic drugs. Anti-FXIIa agents would be valuable to treat or prevent artificial surfaces-related thrombosis such as catheter thrombosis or thrombosis in patients with mechanical heart valves.

Aim: Coumarin derivatives have been previously described as small-molecular-weight inhibitors of FXIIa. Unfortunately, these compounds did not display any activity *in vivo* in a model of thrombosis. In order to discover new chemical entities able to target FXIIa, we launched a study derived from a fragment-based drug design approach. In this study, we evaluated the fragment expansion of a 2,5-dichlorophenyl moiety.

This latter was selected upon the assumption that (1) thrombin and FXIIa should share structural features since they belong to the trypsin-like serine protease family and (2) the 2,5-dichlorophenyl moiety is present in various thrombin inhibitors and is located in the thrombin S1-pocket as demonstrated by crystallographic studies.

Results: About twenty compounds containing a 2,5-dichlorobenzyltriazole scaffold were first obtained by click chemistry. Among these, we identified some displaying an IC_50_ of about 100 μM. Then, we investigated the importance of the 2,5-dichlorophenyl moiety and it appeared that it is needed for the FXIIa inhibition. Finally, we performed modulations on the side chain of the triazole. This afforded compounds with an IC_50_ of about 10 μM. It should also be noted that the most active molecules are selective towards FXIIa.


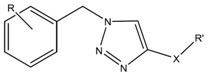


Conclusions & Perspectives: The 2,5-dichlorobenzyltriazole group is a good starting point in the development of small molecular weight inhibitors of FXIIa. Molecular modeling of active compounds would be helpful to better guide the design of more potent FXIIa inhibitors.

### 3.4. Synthesis of New Antimalarial Drugs through an Enantioselective Sharpless Aminohydroxylation Reaction

Guillaume Bentzinger *, Alexandra Dassonville-Klimpt and Pascal Sonnet

Laboratoire LG2A, CNRS FRE 3517, UFR de pharmacie, 1 rue des Louvels, Université Picardie Jules Verne, 80037 Amiens cedex 1, France

* Author to whom correspondence should be addressed; E-Mail: guillaume.bentzinger@u-picardie.fr.

Malaria, due to a *Plasmodium* protozoan, is the 5th most lethal infection in the world (World Malaria Report 2009). The emergence of drug resistance continues to be a serious global problem. New antimalarial drugs are needed and this is why our team is involved in the design and synthesis of new antimalarial compounds. Extensive work has been done to synthesize chloroquine analogs but with much less in regard to mefloquine derivatives. Consequently, mefloquine **1** and its derivatives still remain very attractive synthetic targets. Recently, we have described the asymmetric synthesis and the biological activity of aminoquinolinemethanols **2** (Jonet, A., *et al.* *Tetrahedron Asymmetry* 2011, *22*, 138–148). Some structure-activity relationships have been highlighted: (i) importance of the absolute configuration of asymmetric carbons, (ii) importance of amines (aliphatic *vs*. aromatic). The most active molecule synthesized, in this series, is the aminoquinolinemethanol **3** with a S configuration and a pentyl group (IC_50_ = 6.98 nM) on a chloroquine-resistant W2 strain. We want now study the influence on the biological activity of the position of the amino group and the alcohol group. We present here a synthesis of aminoquinolinethanols **4** through an enantioselective Sharpless aminohydroxylation reaction.


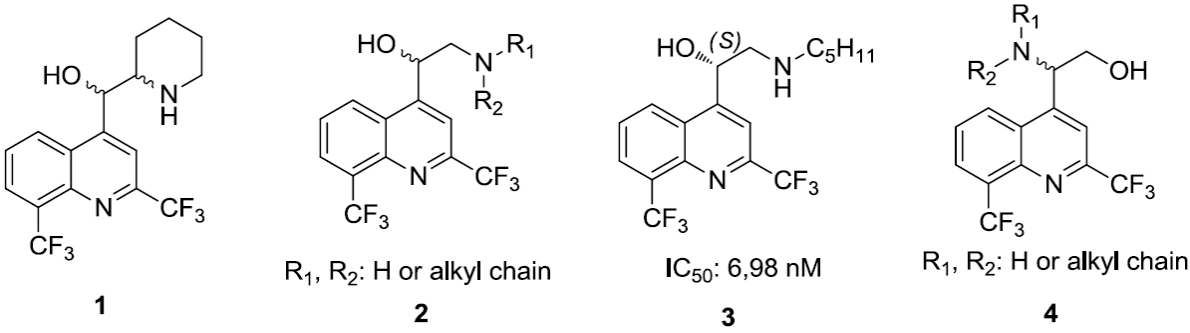


### 3.5. Design, Synthesis and Pharmacological Evaluation of Dimeric Ligands for the Benzothiadiazine Dioxide Allosteric Binding Site of the AMPA Receptors

Thomas Drapier ^1,^*, Eric Goffin ^1^, Christian Krintel ^2^, Ann-Beth Nørholm ^2^, Julien Hanson ^1^, Sébastien Dilly ^1^, Pierre Francotte ^1^, Jette Kastrup ^2^ and Bernard Pirotte ^1^

^1^ Laboratory of Medicinal Chemistry, Center for Interdisciplinary Research on Medicines (CIRM), Université de Liège, Avenue Hippocrate, 15, B36, Liège B-4000, Belgium

^2^ Department of Medicinal Chemistry, Faculty of Pharmaceutical Sciences, University of Copenhagen, Universitetsparken 2, DK-2100, Denmark

* Author to whom correspondence should be addressed; E-Mail: thomas.drapier@ulg.ac.be.

l-Glutamic acid is the major excitatory neurotransmitter in the brain. It exerts its effects through metabotropic and ionotropic receptors. Among the latter, three subtypes have been identified: NMDA, AMPA and KA receptors. It is now well established that a deficit in glutamatergic signaling may be responsible for neurological disorders such as schizophrenia, depression, mild cognitive impairment and ADHD. Enhancement of the signal through positive allosteric modulators of AMPA receptors might be a therapeutic issue for these diseases. These compounds are expected to exert a fine tuning of the signal. Since they require the presence of the endogenous ligand to be active, they are expected to induce less toxicity than agonists.

In this context, based on the structure of known allosteric modulators of AMPA receptors such as cyclothiazide (**1**) and IDRA 21 (**2**), the Laboratory of Medicinal Chemistry (University of Liège) has developed a series of 1,2,4-benzothiadiazine 1,1-dioxides with high potency as AMPA receptor potentiators, among which compounds (**3**) and (**4**). Crystallographic data obtained by the Department of Medicinal Chemistry (University of Copenhagen) highlighted that (**3**) and (**4**) bind to two contiguous sites at the dimer interface of the ligand binding domain of the AMPA receptor (Krintel, C., *et al*. *Biochem. J.* 2012, *441*, 173–178; Nørholm, A.B., *et al.* *J. Med. Chem.* 2013, *56*, 8736–8745). From these data, we may expect that the synthesis of dimeric molecules could lead to further improvement in affinity and activity.

Our work consists in the development of a family of dimeric benzothiadiazine dioxides and their evaluation in a pharmacological assay. Several structural parameters such as the position of the bridge on the aromatic ring between the two heterocycles as well as its nature and length will be studied in order to determine their impact on the activity and thus the affinity.


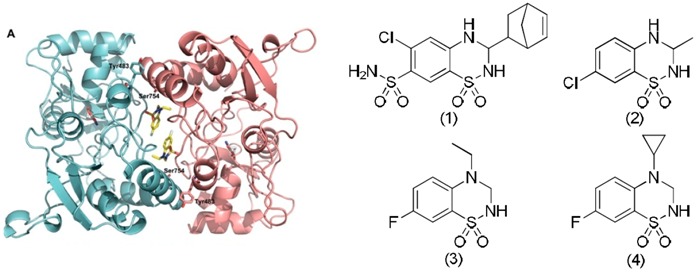


### 3.6. Pyrimido[5,4-d]- and pyrido[3,2-d]-oxazoles: New Original Building Blocks for the Synthesis of Drug Candidates

Lucas Lemaire ^1,^*, Natasha Leleu-Chavain ^1^, Aurélien Tourteau ^1^, Alaa Abdul-Sada ^2^, John Spencer ^2^ and Régis Millet ^1^

^1^ University Lille Nord de France, ICPAL, Inserm U995-LIRIC, Lille 5900, France

^2^ University of Sussex, Falmer, BN1 9QJ, UK

* Author to whom correspondence should be addressed; E-Mail: lucas.lemaire@univ-lille2.fr.

Heterocycles are important scaffolds in drug design. Therefore, the development of efficient synthesis routes to new heterocycles is crucial in medicinal chemistry. Especially, oxazole derivatives have been increasingly used for the synthesis of biological compounds and are present in many drugs, as antibiotics, anti-inflammatory, or antiproliferative compounds for example.

5-Aminooxazole-4-carbonitrile is an interesting bifunctional building block for drug design and organic synthesis. Indeed, the enaminonitrile moiety is well known to be highly reactive and can be used for the synthesis of various heterocycles, including not easily available heteroaromatics. Moreover, the 5-aminooxazole-4-carbonitrile scaffold can be obtained through a one pot reaction (Spencer, J., *et al.* *Tetrahedron Lett.* 2012, *53*, 1656–1659).

The aim of our work was to synthesize various heterocycles in short synthesis routes using 5-amino-oxazole-4-carbonitrile as a common starting building block. From this scaffold, 10 different pyrimido[5,4-d]oxazoles and 4 pyrido[3,2-d]oxazoles were synthesized in one or two steps (Lemaire, L., *et al*. *Tetrahedron Lett.* 2015, *56*, 2448–2450). Synthetic procedures have been adapted to overcome the instability of the oxazole. These heterocycles will be substituted with suitable groups in order to develop and synthesize potential CB2 agonist ligands.


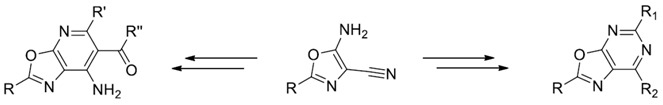


### 3.7. Design, Synthesis and Characterization of 2-Methylpyridine Substituted Harmine Derivatives as Anticancer Compounds

Sébastien Marx

Namur Medicine & Drug Innovation Center (NAMEDIC), Laboratory of biological chemistry (CBS), University of Namur (Unamur), 61 rue de Bruxelles, Namur 5000, Belgum; E-Mail: sebastien.marx@student.unamur.be

Harmine and its derivatives are largely studied for their cytotoxic and antitumor effects (Khan, F., *et al.* *Eur. J. Pharmacol.* 2013, *721*, 391–394). Hence, new trisubstituted derivatives were designed and synthetized, in particular compound 1 has shown a submicromolar anticancer activity (Meinguet, C. MSc. Thesis, Université du droit et de la santé de Lille II, Lille, France, 2012; p. 71; Frédérick, R., *et al.* *J. Med. Chem.* 2012, *55*, 6489–6501).

Because molecule **1** presents a good anticancer activity, *in vitro* tests have been performed and have shown that this compound gave a lower toxicity on healthy cells than on cancer cells. Moreover, *in vivo* toxicity has been determined by a maximal tolerated dose study (1 mg/kg). Nevertheless, compound **1** has shown an average solubility (410 μM) that leads to the formation of a suspension and not a solution for *in vivo* tests. So, it has to be administrated by intraperitoneal injection which leads a lower absorption than intravenous injection.


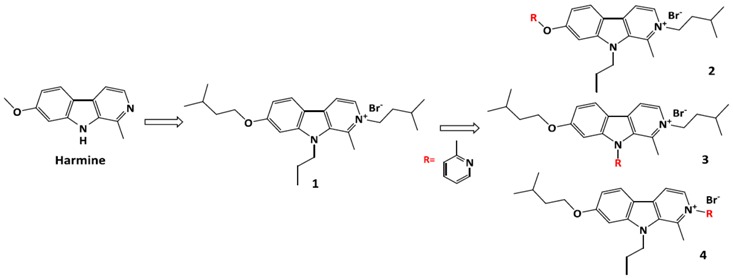


The objective of this work consists of synthesizing derivatives **2**, **3**, **4** including a 2-methylpyridine substituent of compound **1** in order to increase solubility with pyridine moiety and keeping anticancer activity. To achieve this goal, the adopted strategy was to perform a theoretical study of designed molecules in order to predict lipophilicity (logP) and anticancer activity with a CoMFA model. These derivatives are predicted to be more hydrophilic than compound **1** and as active as it. Based on these results, derivatives **2**, **3** and **4** were synthesized and characterized. Compound **4** was obtained by mechanochemistry and has shown a submicromolar activity such as compound **1**. As a perspective, the solubility of molecule **4** will be measured in injection solution for *in vivo* tests.

### 3.8. A New Concept of Dual Targeting Drugs: DTP-348 a Carbonic Anhydrase IX Inhibitor Specific for Hypoxic Tumors

Parvathaneni Nanda Kumar ^1,2^, Ludwig Dubois ^2^, Sarah G.J.A. Peeters ^2^, Simon J.A. van Kuijk ^2^, Ala Yaromina ^2^, Guiseppina De Simone ^3^, Claudia T. Supuran ^4^, Philippe Lambin ^2,^* and Jean-Yves Winum ^1,^*

^1^ Institut des Biomolécules Max Mousseron (IBMM) UMR 5247 CNRS-­-ENSCM Université de Montpellier, ENSCM 8 rue de l’EcoleNormale, Montpellier 34296, France

^2^ Department of Radiation Oncology (MAASTRO Lab), GROW—School for Oncology and Developmental Biology, Maastricht University Medical Centre, Universiteitssingel 50/23, PO Box 616, Maastricht 6200 MD, The Netherlands

^3^ Istituto di Biostrutture e Bioimmagini-CNR, via Mezzocannone 16, Naples 80134, Italy

^4^ Department of Chemistry, Laboratory of Bioinorganic Chemistry, Università degli Studi di Firenze, Firenze 048017, Italy

* Author to whom correspondence should be addressed; E-Mail: jean-yves.winum@univ-montp2.fr.

Hypoxia is one of the most devastating phenomenons while treating remote and solid tumors by radio/chemotherapies. pH regulating transmembrane carbonic anhydrase IX is associated with poor prognosis and therapy resistance. This made carbonic anhydrase IX as potential anticancer therapeutic target (McDonald, P.C., *et al. Oncotarget* 2012, *3*, 84–97).

We developed series of nitroimidazoles incorporating sulfamide/sulfonamide/sulfamate moieties were having radio/chemo sensitization property to target tumor-associated carbonic anhydrase isoforms IX and XII (Dubois, L., *et al.* *Radiother. Oncol.* 2013, *108*, 523–528). Most of the new compounds were nanomolar inhibitors of these isoforms. Inhibition efficacy of these molecules strongly suggested by crystallographic studies on adduct of DTP-348 with hCAII. By reducing hypoxia-induced extra cellular acidosis in HT-29 and HeLa cell lines these molecules showed significant activity of CAIX inhibition, this was shown by crystal structure (*in vitro*) (Rami, M., *et al.* *J. Med. Chem.* 2013, *56*, 8512–8520).


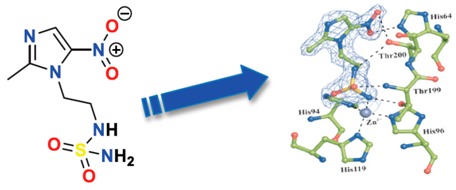


The lead molecule in sulfamide series (DTP-348) showed chemosensitization co-treated with Doxorubicin and radiosensitization (*in vivo*) of carbonic anhydrase IX containing hypoxic tumors. We were motivated to proceed with pre-clinical trials on DTP-348 with encouraging ADME (*in vitro*) screening results, pharmacokinetic and cytotoxicity data (Dubois, L., *et al.* WO 2012/087115, 28 June 2012) (Table 1).

**Table 1:** Pharmacokinetics data for compounds tested.

Compd.	Solubility Bound (µM)	Permeability Efflux Ratio	Stability Clearance	Metabolism Cytochrome Inhibition (µM)	Plasma Protein Binding Mean Fraction Unbound
DH338	>100	1.11	6.67	>25	0.422
DTP348	>100	0.638	4.88	>25	1.000
NKP60	>100	1.11	2.27	>25	0.308

## 4. Posters

### 4.1. Pharmacology and Signaling Pathways of the Orphan GPR85/SREB2

Céline Laschet ^1,2,^*, Nadine Dupuis ^1,2^, Anouar Derj ^1,2^, Julie Gilissen ^1,2^, Pierre Geubelle ^1,2^, Arvind Soni ^1,2^, Bernard Pirotte^2^ and Julien Hanson ^1,2^.

^1^ ULG, GIGA-Molecular Pharmacology, CHU, tour GIGA (+4), 15, av Hippocrate, Liège 4000, Belgium

^2^ ULG, CIRM-Medicinal Chemistry, CHU, B36 (+5), 15, av Hippocrate, 4000 Liège, Belgium

* Author to whom correspondence should be addressed; E-Mail: celine.laschet@ulg.ac.be.

GPCRs are the largest family of membrane receptors and are characterized by seven transmembrane domains. This family of receptors is currently the most successfully targeted protein for therapeutic purposes (Rask-Andersen, *et al.* *Nat Rev Drug Discov.* 2011, *10*, 579–590**)**. In the presence of an activating ligand, heterotrimeric G proteins will bind to the receptor. This process will lead to separation of the Gα-subunit and the Gβγ-complex. Both will modulate the activity of a variety of effectors (Audet, *et al*. *Cell*, 2012, *151*, 14–23). The extinction of G protein signaling is processed by phosphorylation of the C-terminal tail of the receptor. Phosphorylation and activation enhances the affinity of the receptor for the cytosolic adaptor proteins β-arrestins that promote receptor internalization (Shenoy, *et al.* Science, 2001, *294*, 1307–1313).

GPR85 is a member of the Superconserved Receptor Expressed in the Brain (SREB) subfamily, a unique cluster of 3 receptors. It has the rare characteristic of sharing 100% sequence homology with its mouse and rat homologs. No ligands have been proposed for SREB2/GPR85 and it is considered orphan (Hellebrand, *et al.* *Brain Res Gene Expr Patterns* 2001, *1*, 13–16). Its mRNA has been detected in olfactory bulb, whole eye, Pituitary gland, islets of Langerhans, Ghrelin cells, muscle-myenteric nerve layer and macrophages (Regard, *et al.* *Cell* 2008, *135*, 561–571; Engelstoft, *et al.* *Mol MeTable* 2013, *2*, 376–392; Ito, *et al.* *Cell Tissue Res.* 2009, *338*, 257–269; Lattin, *et al.* *Immunome Res.* 2008, *4*, 5).It has also been detected in all neurons of higher brain structures and more specifically in regions characterized by high levels of neuronal plasticity. GPR85 has been shown to negatively influence brain size, impair hippocampal adult neurogenesis, neurogenesisdependent learning and memory behavior. An increase of vulnerability to schizophrenia was also linked to genetic variations of GPR85 human gene (Matsumoto, *et al.* *PNAS* 2008, *105*, 6133–6138).

Our research project proposes to investigate the molecular pharmacology of GPR85. The strategy is to characterize precisely GPR85 signaling pathways in a ligand independent manner (G protein, Arrestins, trafficking, ERK, c-Src,...). In addition, we plan to explore GPR85 specific features compared to archetypal rhodopsin-like GPCRs and the interplay with other SREB members. Knowing how the receptor behaves at a molecular and cellular level may lead to a better understanding of GPR85 functions reported in literature. Moreover, an accurate vision of the receptor molecular pharmacology will pave the way to the ultimate goal of discovering its endogenous ligands.

### 4.2. Ligand-Based Pharmacophore Modeling and Virtual Screening for the Discovery of Novel Myeloperoxidase Inhibitors

Jalal Soubhye ^1,^*, Iyas Aldib ^1^, Paul G. Furtmüller ^2^, Jean Nève ^1^, Christian Obinger ^2^, François Dufrasne ^1^ and Pierre Van Antwerpen ^1^

^1^ Laboratoire de Chimie Pharmaceutique Organique, Faculté de Pharmacie, Faculty of Pharmacy, Université Libre de Bruxelles, Brussels, Belgium

^2^ Department of Chemistry, Division of Biochemistry, Vienna Institute of BioTechnology, BOKU – University of Natural Resources and Life Sciences, Vienna, Austria

* Author to whom correspondence should be addressed; E-Mail: jal.sub@hotmail.com.

The most important problem for the medicinal chemist in the development of new drugs is to find good hits. Molecular informatics virtual screening is one of the most important rational screenings. The immunity enzyme MPO is one of the new targets for finding novel antiinflammatory agents. Because the structure of this enzyme was determined and the enzyme was crystallized with several ligands, some medicinal chemists used docking programs in order to discovery new MPO inhibitors (Malvezzi, A., *et al.* *Mol. Inform.* 2011, *30*, 605–613; Aldib, I., *et al.* *J. Med. Chem.* 2012, *55*, 7208–7218).

In our research, four filters were applied in order to select several compounds to be tested *in vitro* as MPO inhibitors. 727842 Compounds of the Zinc database were filtered on Lipinski’s rule explorer which selects only the compounds that have molecular properties important for a drug’s pharmacokinetics in the human body. Then, the best inhibitors that are described in the papers were taken as templates for the ligand-based pharmacophore screening. This screening was achieved by LigandScout 3.12 Software which selects the compounds that have the same chemical groups of the templates. Four models were taken as templates where each model consists of two inhibitors. The resulting compounds were subjected to a docking program (LeadIT 2.1 Software). The compounds that feature stacking pose on the heme of the active site and achieve free energy of interaction with the active site less than ‒8 Kcal were taken. Fifty nine compounds passed this filter (24 compounds from model 1, 20 compounds from model 2, 12 compounds from model 3 and three compounds from model 4). OSIRIS Property Explorer was applied to the obtained molecules. This program calculates toxicity risk, cLogP, solubility, molecular weights and drug-likeness in order to select the compounds that have high Overall Drug-Likeness Score where only 30 compounds passed this filter. These 30 compounds were tested *in vitro* and the IC_50_ values were determined. Twelve hits were found to have IC_50_ values of less than 5 μM and three of them have activity at nanomolar range. A kinetic study was done for the active and non-active compounds to determine the mechanism of inhibition of the good inhibitors and the reasonsof losing the activity of the non-active compounds in spite of the high affinity. In conclusion, using ligand-based pharmacophore screening in addition to the docking screening test, several new MPO inhibitors were obtained. These inhibitors can be used as hits for developing high potent inhibitors.


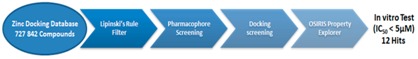


### 4.3. Design, Synthesis, Characterization, Pharmacological Evaluation and in Vivo Studies of Harmine Derivatives as New Anticancer Compounds

Céline Meinguet ^1,^*, Raphaël Frederick ^2^, Céline Bruyère ^3^, Véronique Mathieu ^3^, Julie Laloy ^1^, Gerhard Wolber ^4^, Robert Kiss ^3^, Bernard Masereel ^1^ and Johan Wouters ^1^

^1^ Namur Medicine & Drug Innovation Center (NAMEDIC-NARILIS), University of Namur (Unamur), 61 Rue de Bruxelles, 5000 Namur, Belgium

^2^ Medicinal Chemistry Research Group (CMFA), University of Louvain (UCL), 73, avenue Mounier, 1200 Bruxelles

^3^ Laboratoire de Toxicologie, Faculté de Pharmacie, Université libre de Bruxelles (ULB), Boulevard du Triomphe, 1050 Brussels, Belgium

^4^ Institute of Pharmacy, Freie Universität Berlin , 2+4 Königin Luise Straβe, 14195 Berlin, Germany

* Author to whom correspondence should be addressed; E-Mail: celine.meinguet@unamur.be.

Harmine is a natural β-carboline known to be associated with anticancer activity by inhibiting diverse enzymes. Recently, we found that trisubstituted β-carboline derivatives were characterized with potent *in vitro* anticancer properties using an MTT assay on 3 glioma and 2 esophageal cancer cell lines. These molecules are substituted on the 2,7 and 9-positions, by a large range of apolar substituents (Frédérick, R., *et al. J. Med. Chem.* 2012, *55*, 6489–6501).


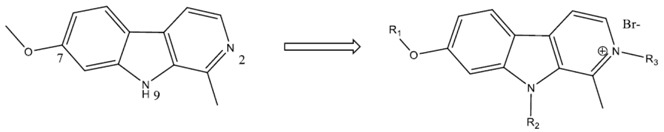


Despite an antiproliferative activity in the micromolar and submicromolar range and a cytostatic profile on cancer cells, some of these molecules have a poor solubility. In order to obtain novel druggable harmine-based antiproliferative molecules, and following the concept of early-ADME which leads to improve both biological and ADME properties, the goal of this work was to increase the solubility of the β-carboline derivatives preserving their antiproliferative activity on cancer cells.

To achieve this goal, the adopted strategy was to develop a 3D-QSAR model which indicates the important structural characteristics for biological activity. Based on this model, several molecules were synthesized. Results show that synthesized molecules are more soluble than the first set of molecules while maintaining a micromolar to submicromolar activity (Meinguet, C., *et al. Eur. J. Med. Chem*. 2015, *94*, 45–55).

Based on these promising results, a pharmacological study of the best antiproliferative compound was led. Results underline an *in vitro* selectivity for cancer cells compared to healthy cells. Moreover, maximal tolerated dose study on mice has highlighted no toxicity at 1mg/kg. Based on a 3D-QSAR model, new compounds were synthesized. These compounds combine high solubility at physiological pH and micromolar to submicromolar activity on diverse cancer cells. Pharmacological evaluation of the best compound underline *in vitro* selectivity for cancer cells compared to healthy cells and no *in vivo* toxicity at 1 mg/kg. As a perspective, we will study the *in vivo* anticancer action.

### 4.4. Targeting DNA methylation as epigenetic therapy for adult T-cell leukemia/ lymphoma (ATLL)

Grégoire Rondelet ^1,^*, Luca Willems ^2^ and Johan Wouters ^1^

^1^ University of Namur, Drug Design and Discovery Center, Rue de Bruxelles, 61, Namur B-5000, Belgium

^2^ Université de Liège, National Fund for Scientific Research, Molecular Biology, GIGA and Gembloux Agro-Bio Tech, Liège 4000, Belgium

* Author to whom correspondence should be addressed; E-Mail: gregoire.rondelet@unamur.be.

Viruses are responsible for 15%–20% of human cancers worldwide. Among them, the human T-cell leukemia/lymphoma oncovirus type I (HTLV-I) is associated with adult T-cell leukemia and lymphoma (ATLL) (Manns, A., *et al*. *Lancet* 1999, *353*, 1951–1958). The main obstacle in the treatment of this cancer is the absence of viral expression, thereby escaping recognition by the immune system and propagation of the transformed cells (Merimi, M., *et al*. *J. Virol.* 2007, *81*, 5929–5939). Indeed, epigenetic modifications as hypermethylation of particular DNA promoters, catalyzed by DNA (cytosine-5) methyltransferases (DNMTs), lead to transcriptional silencing of tumor suppressor genes (Hatta, Y., *et al.* *Leukemia* 2002, *16*, 1069–1085). Therefore, inhibitors of DNMTs yield novel opportunities in this cancer research field (Rodríguez, S.M., *et al.* *Viruses* 2011, *3*, 1210–1248).


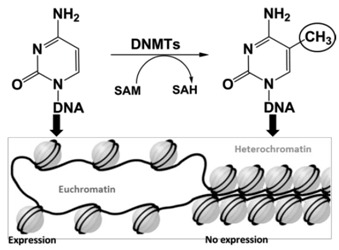


The aim of the present work is the identification and characterization of ligands for the regulatory domain of human enzymes DNMT3A and DNMT3B, the domain PWWP. PWWP domains interact with both DNA and histones and are important to establish the methylation pattern. A study of 2011 has solved for the first time the structure of the complex of PWWP domains of both Dnmt3A (PDB code 3LLR) and Dnmt3B (PDB code 3QKJ) with a ligand, the bis-tris molecule (Wu, H., *et al.* *PLoS ONE* 2011, *6*, e18919). The presented strategy to identify novel lead compounds is based on similarity-based virtual screening of bis-tris molecule. Up to now, we have resolved structures of dnmt3b PWWP domain in complex with different bis-tris analogs. These analogs could inhibit the DNMT3A/3B-histone interaction to re-express the tumor suppressor genes.

### 4.5. Crystallographic Study of the Mechanisms of Inhibition of IDO and TDO Enzymes through Protein Engineering

Quentin Thémans * and Johan Wouters

Laboratoire de Chimie Biologique Structurale (CBS), University of Namur (UNamur), 61 rue de Bruxelles, 5000 Namur, Belgium

* Author to whom correspondence should be addressed; E-Mail: quentin.themans@unamur.be.

Cancer is a leading cause of death in the world, especially in developed countries. To fight against it, a therapeutic arsenal has been developed including cancer immunotherapy, a recent and promising option (Mapara, M.Y., *et al. J. Clin. Oncol.* 2004. *22*, 1136–1151; Ernst, B., *et al. Curr. Oncol. Rep.* 2015, *17*, 1–10).

The enzymes indoleamine 2,3-dioxygenase (hIDO) and tryptophan 2,3-dioxygenase (hTDO) catalyze the dioxygenation of L-tryptophan to form N-formylkynurenine and therefore regulate the levels of tryptophan in the body. Overexpression of these enzymes in many cancers causes a local depletion of tryptophan which induces tolerance to the host immune system and allows tumor cells to avoid immune rejection (Ernst, B., *et al. Curr. Oncol. Rep.* 2015, *17*, 1–10; Opitz, C.A., *et al.* *Nature* 2011, *478*, 197–203). Therefore, these enzymes are attractive targets in the context of anti-cancer immunotherapy.

The aim of this project is to identify and develop inhibitors of human enzymes hIDO and hTDO through a better understanding of the catalytic and inhibition mechanisms of these systems at the molecular level. Crystallography is the method of choice to achieve this objective. It will be combined with enzymatic characterizations and mutagenesis to obtain high resolution crystallographic structures in complex with inhibitors (ex: LM10) (Pilotte, L., *et al.* *PNAS*, 2012. *109*, 2497–502).


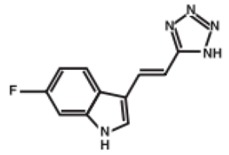


To achieve this ambitious objective, we will use molecular engineering through the construction of mutants of surface to facilitate the crystallization of enzymes (Derewenda, Z.S. *Structure* 2004, *12*, 529–535; Derewenda, Z.S. *Acta Cryst.* 2011 D67, 243-248). These new constructs will then be used to determine the structures of enzyme-inhibitor complexes with high resolution. A series of mutations in the active site will also be created for a better understanding of the mechanism and the substrate specificity of the two enzymes. This approach will help in the design of mixed inhibitors of hIDO and hTDO.

### 4.6. Design and Synthesis of PET-Probes Targeting AMPA Subtype Receptors

François Deverdenne ^1,^*, Gisèle Claes ^1^, Eric Goffin ^1^, Alain Plenevaux ^2^, Bernard Pirotte ^1^, André Luxen^2^ and Pierre Francotte ^1^

^1^ Laboratory of Medicinal Chemistry, Center for Interdisciplinary Research on Medicine, Université de Liège, Av. Hippocrate, 15, B36, Liège 4000, Belgium

^2^ Cyclotron Research Center, Université de Liège, Allée du 6 Août, B30, Liège 4000, Belgium

* Author to whom correspondence should be addressed; E-Mail: fdeverdenne@ulg.ac.be.

The AMPA subtype of glutamatergic receptors is the main actor in the fast excitatory neurotransmission in the mammalian central nervous system. These receptors are involved in the expression and the maintenance of the long-term potentiation, a phenomenon closely linked to cognitive and memorization processes. Based on experimental data, it also appears that glutamatergic systems are involved in several pathological diseases. For instance, a lack of glutamatergic neurotransmission is observed in cognitive disorders or schizophrenia and an excessive activity is observed in Parkinson or Huntington diseases.

The *in vivo* study of glutamate receptors mapping and its evolution appears to be an essential step for a better understanding of its implications. However, according to the literature, design of such a probe remains difficult due to the lack of specificity of the probes.

Taking into account the potential *in vitro* and *in vivo* activity and specificity of benzothiadizine dioxides (BTDs) acting as AMPA positive allosteric modulators, we are investing the development of new compounds of this class radiolabeled with a fluorine-18 atom. Hence, we are currently developing new series of BTDs characterized by the presence of a fluorine atom and a 7-phenoxy-substituent that are expected to be more active and more specific. Finally, pharmacological tests to evaluate the best candidates for the radiochemical synthesis and *in vivo* evaluations are currently in progress.

### 4.7. Synthesis and Study of Siderophore Analogues as Antimicrobial Agents

Marine Pillon *, Alexandra Dassonville-Klimpt, Emmanuel Baudrin, Guillaume Bentzinger and Pascal Sonnet

LG2A CNRS FRE 3517, Axe Chimie pour le Vivant, Faculté de Pharmacie 1 rue des Louvels, 80037, AMIENS Cedex

* Author to whom correspondence should be addressed; E-Mail: marine.pillon@u-picardie.fr.

Iron is an essential element for the growth and the survival of organisms (transport of oxygen by hemoglobin, cofactor of ribonucleotide reductase). Siderophores are natural iron chelators which possess a very high affinity to ferric iron such as rhodotorulic acid (produced by *Rhodotorula pilimanæ*), dimerumic acid (*Scedosporium apiospermum*) or pyochelin (*Pseudomonas aeruginosa*). Siderophores can be potentially used for a wide variety of therapeutic applications. For example, they can be coupled with an antibiotic and use in “Trojan horse” strategy, as iron detoxification agent, antioxidant, antimicrobial or anticancer drugs. We present here the synthesis, physicochemical properties and antimicrobial studies of different siderophore analogues.

### 4.8. Discovery of Donecopride: A Novel Multi-Target Directed Ligand for Alzheimer Disease

Christophe Rochais ^1^, Cédric Lecoutey ^1^, Thomas Freret ^2^, Céline Ballandonne ^1^, Valentine Bouet ^2^, Patrizia Giannoni ^3^, Florence Gaven ^3^, Sylvie Claeysen ^3^, Michel Boulouard ^2^ and P. Dallemagne ^1,^*

^1^ Centre d’Etudes et de Recherche sur le Médicament de Normandie (CERMN) - UPRES EA 4258 - FR CNRS INC3M - SFICORE, Université de Caen Basse-Normandie, UFR des Sciences Pharmaceutiques - Bd Becquerel, France

^2^ Groupe Mémoire et Plasticité comportementale (GMPc) - EA4259 - Université de Caen Basse-Normandie, UFR des Sciences Pharmaceutiques - Bd Becquerel, F-14032 Caen, France

^3^ CNRS, UMR-5203, Institut de Génomique Fonctionnelle, F-34000 Montpellier, France

* Author to whom correspondence should be addressed; E-Mail: patrick.dallemagne@unicaen.fr.

Complex pathologies such as Alzheimer's disease (AD) would benefit from a combination of actions targeting, in the same time, several molecular causes implied in the pathogenesis. Our aim was to design a multi target-directed ligand (MTDL) gathering two properties: acetylcholinesterase (AChE) inhibition and 5-HT4 receptor activation. AChE inhibition is the action mechanism of donepezil, the current available drug for AD. Activation of 5-HT4 receptors promotes the nonamyloidogenic cleavage of the amyloid precursor protein (APP) and the release of the neuroprotective soluble APPalpha fragment. Combining a dual-binding site pharmacophore of AChE inhibitors with a pharmacophore of 5-HT4R ligands, we isolated a candidate compound and performed pharmacomodulation of this hit to optimize its properties. We selected donecopride1 as a druggable lead able to inhibit AChE (IC_50_ = 16 nM) and to induce sAPPalpha release (EC_50_ = 11.3 nM) upon 5-HT4R activation (K_i_ = 10.4 nM; 48.3% of serotonin response). *In vivo* properties of this new compound in the 5XFAD mouse model of AD (acute and chronic administration) will be presented (Lecoutey, C., *et al.* *PNAS* 2014, *111*, E3825–E3830).

### 4.9. Continuous-Flow Synthesis of Indole Derivatives and Reusable Supported Palladium Catalyst to Sonogashira Reaction

Joseph D'Attoma ^1,^*, Grégoire Cozien ^1^, Pierre L. Brun ^3^, Yves Robin ^3^, Stéphane Bostyn ^2^, Frédéric Buron ^1^ and Sylvain Routier ^1^

^1^ Institut de Chimie Organique et Analytique (ICOA), Université d’Orléans, UMR CNRS 7311, Rue de Chartres, BP 6759, 45067 Orléans, France

^2^ Institut Universitaire Technologique (IUT) de Chimie, Université d’Orléans, 16 Rue d'Issoudun, BP 16729, 45067 Orléans Cedex 2, France

^3^ ISOCHEM, 4 rue Marc Sangnier, BP 16729, Pithiviers 45300, France

* Author to whom correspondence should be addressed; E-Mail: joseph.dattoma@univ-orleans.fr.

Indole is an aromatic heterocycle present in many structures of natural alkaloids and in a multitude of biologically and pharmacologically active compounds. For example, it is found as the structural scaffold of signaling mediators or hormones such as melatonin (Taber, D.F., *et al.* *Tetrahedron* 2011, *67*, 7195–7210). Given the interest generated by this heterocyclic compound, it is not surprising that fast and efficient access to these derivatives remains of interest to the chemical community (Merkul, E., *et al.* *Chem. Rev.* 2007, *107*, 2300–2318). To functionalize indole and its derivatives, we were interested in the development of an innovative technology in organic chemistry, continuous flow synthesis. This eco-efficient system offers a variety of advantages like faster and cleaner reactions with less solvent as well as the possible manipulation of short-lived and highly reactive species. Benefits also include diminished chemical exposure and easy scale-up to furnish larger amounts of product necessary for *in vivo* studies. The aim of this project is to transfer usual reactions of indole chemistry to continuous flow process. Thus, this communication will present this transposition for C-3 iodination and NH protection with BOC group. Moreover, we investigated an original copper-free Sonogashira reaction in C-3 position using supported Pd catalyst.


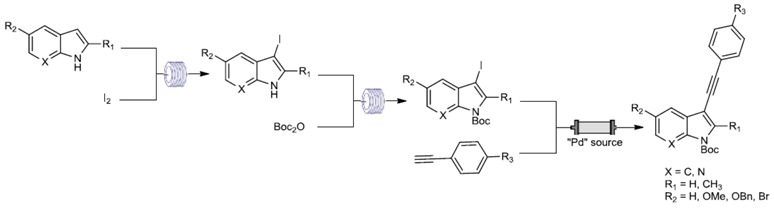


## 5. Conclusions

At the end of the meeting, the following prizes were awarded:

- Prize for the best oral communication: A. Ameryckx of the University of Louvain (Belgium) for her talk “Design and synthesis of DD-ligases inhibitors: new targets for potential antibiotics”.
- Prize for the best poster presentation: A. Derj of the University of Liège (Belgium) for his poster “Unraveling the signaling and dimerization properties of CXCR3 isoforms and their interlay with CXCR7 in the context of glioblastoma pathology”.
- Prize of the Journées Franco-Belges de Pharmacochimie. This prize is awarded to a young scientist (young doctor or ending their PhD degree) with an outstanding curriculum vitae, to encourage them to pursue a career in science. This year the prize goes to S. Ravez of the University of Louvain (Belgium).

In 2016 the “30ièmes Journées Franco-Belges de Pharmacochimie” will be held in France.

